# Supplementary material for: Portuguese validation of the Alcohol Craving Questionnaire–Short Form–Revised
Source: PLoS One. 2021 May 24;16(5):e0251733. doi: 10.1371/journal.pone.0251733 (PMC8143387; doi:10.1371/journal.pone.0251733)
Supplement: S1 Appendix — (PDF) [file pone.0251733.s001.pdf]

Questionário de Craving pelo Álcool - Versão Curta - Revista

INSTRUÇÕES: Por favor indique o quanto concorda ou discorda com cada uma das seguintes afirmações, colocando uma cruz (X) em cada uma das linhas entre discordo totalmente e concordo totalmente. Quanto mais próximo colocar a cruz de uma extremidade ou de outra indicará a força da sua discordância ou concordância. Temos interesse em saber o que está a pensar ou a sentir neste preciso momento em que preenche este questionário. Por favor preencha todos os itens.

NESTE MOMENTO

1. Se eu tivesse álcool provavelmente bebê-lo-ia

DISCORDO TOTALMENTE___:___:___:___:___:___:___CONCORDO TOTALMENTE

2. Sinto falta de beber.

DISCORDO TOTALMENTE___:___:___:___:___:___:___CONCORDO TOTALMENTE

3. Não estou a planear beber.

DISCORDO TOTALMENTE___:___:___:___:___:___:___CONCORDO TOTALMENTE

4. Não conseguiria evitar beber se tivesse álcool aqui.

DISCORDO TOTALMENTE___:___:___:___:___:___:___CONCORDO TOTALMENTE

5. Quero tanto beber que quase sinto o sabor do álcool.

DISCORDO TOTALMENTE___:___:___:___:___:___:___CONCORDO TOTALMENTE

6. Sentir-me-ia menos irritável se bebesse álcool agora.

DISCORDO TOTALMENTE___:___:___:___:___:___:___CONCORDO TOTALMENTE

7. Se bebesse álcool, sentir-me-ia menos tenso(a).

DISCORDO TOTALMENTE___:___:___:___:___:___:___CONCORDO TOTALMENTE

8. Beber não seria muito satisfatório.

DISCORDO TOTALMENTE___:___:___:___:___:___:___CONCORDO TOTALMENTE

9. Sentir-me-ia menos inquieto(a) se bebesse álcool.

DISCORDO TOTALMENTE___:___:___:___:___:___:___CONCORDO TOTALMENTE

10. Se estivesse a beber álcool, sentir-me-ia menos nervoso(a).

DISCORDO TOTALMENTE___:___:___:___:___:___:___CONCORDO TOTALMENTE

11. Seria fácil deixar passar a oportunidade de beber álcool.

DISCORDO TOTALMENTE___:___:___:___:___:___:___CONCORDO TOTALMENTE

12. Beber colocar-me-ia de melhor humor.

DISCORDO TOTALMENTE___:___:___:___:___:___:___CONCORDO TOTALMENTE
