# Supplementary material for: Portuguese validation of the Alcohol Craving Questionnaire–Short Form–Revised
Source: PLoS One. 2021 May 24;16(5):e0251733. doi: 10.1371/journal.pone.0251733 (PMC8143387; doi:10.1371/journal.pone.0251733)
Supplement: Attachment — Submitted filename: Response to Reviewers.docx [file pone.0251733.s003.docx]

Dear Dr. Wen-Jun Tu,

We sincerely thank you for inviting us to resubmit a revised version of our manuscript. We have considered each of the reviewers’ suggestions very carefully, and we have rephrased the parts of the manuscript pointed out by them, as specified below. We believe that all the referees’ concerns have been addressed in this new version of the manuscript, and we hope it can now be considered suitable for publication on PLOS ONE.

Please, do not hesitate to contact us for further information or if any other questions come up.

Thank you for your time and consideration.

We look forward to hearing from you.

We thank the reviewers for their useful suggestions. In the next pages, we try to address each of their considerations.

**Reviewer 1**

First of all, we thank the reviewer for their valuable comments and suggestions as we believe they have helped substantially to improve the manuscript.

***Issue 1. Writing corrections***

1. In the abstract, “college” was added before “participants” for a better understanding and more specific characterization of the sample.
2. On page 5 where it showed “67,3%” has been changed to “67.3%”, in accordance with the correct system of decimals. All other decimals have been checked.
3. Also, on page 5, “8” has been changed to “eight”, to meet the standard professional rules for use of numerals. Additionally, the manuscript was checked for similar cases.
4. On page 8, “to this extend” has been changed to “to this extent”.
5. On page 12, “for a by gender factor analysis” has been changed to “for a factor analysis by gender” and “similar variances” has been changed to “similar amounts of variance”.
6. On page 13, “this scale also allows to evaluate” has been changed to “this scale also allows one to evaluate”.
7. On page 14, “which previously was comprised in the Compulsivity dimension,” has been changed to “which previously loaded on the Compulsivity factor”; “Compulsivity factor was composed by” has been changed to “Compulsivity factor was composed of”; “ability to restrain from” has been changed to “ability to refrain from”.
8. On page 15, “version” has been changed to “study” and “who have” has been changed to “so they had”; “As such” was removed from the sentence; “changes on craving levels” has been changed to “changes in craving levels”; “fulfilled” has been changed to “completed”.
9. On page 16, “Concluding” has been changed to “In conclusion”.
10. The phrase “having a short extension” was reworked and changed to “having a shorter length”.

Additionally, the manuscript was revised by an independent person with high proficiency in English and further changes were made regarding grammar.

***Issue 2. Clarification concerning the use of European or Brazilian Portuguese when referring to the sample and validation language***

The authors would like to thank the Reviewer 1 for highlighting these issues since it could, in fact, lead the reader towards misconceptions regarding the nature of the study and its objectives. In this sense, the manuscript was fully reviewed, and expressions “Portuguese citizens” and “European Portuguese speakers” were introduced to provide more clarity.

Despite it remains true that the Brazilian validation of ACQ-SF-R was developed in Portuguese, there are differences between Brazilian Portuguese and European Portuguese. Additionally, the need for a validation in European Portuguese was also tied in with the sociocultural differences between Brazilian and Portuguese citizens. In this sense, this is, indeed, the first validation of the scale for the population of Portugal, while also being the first in European Portuguese.

***Issue 3. Inaccurate statements suggesting the scale validation in a clinical sample***

We thank the reviewer for bringing our attention to these inaccurate statements. All mentions of the use of a clinical sample, or possible validation of the scale in a clinical population have been removed from the manuscript. Additionally, in the end of the discussion (pp. 16, lines 325 and 335) we suggested that future investigation should include a clinical sample aiming to strengthen the scale’s generalization properties, while also enabling its use in these populations.

“Although part of the sample had a risky consumption pattern, participants cannot be considered a clinical population. As such, results from this validation should not be generalized to clinical populations (e.g., patients with AUD)”.

“Further research including a greater sociodemographic representation as well as the inclusion of a clinical (i.e., alcohol-dependent) group is recommended in order to enhance the external strength and generalization properties of the questionnaire (e.g., for using in clinical setting).”.

***Issue 4. Confusing sentence regarding ACQ-SF-R being a complement to PACS***

As it was highlighted by reviewer, the sentence “The present study seems to complement the only Portuguese-validated questionnaire so far assessing craving in clinical and non-clinical population” was indeed confusing. In this sense, the full sentence was amended to “Thus, the present study seems to complement the PACS, which to date is the only questionnaire validated in European Portuguese assessing alcohol craving” providing a better understanding of its true meaning. In this sense, what the sentence should imply is that ACQ-SF-R comes to fill possible gaps that PACS had (e.g., only having a single factor), and together they can mutually enhance each other.

Nevertheless, if the use of the word “complement” remains confusing or misused we will gladly change it for another that fits better in the meaning of the sentence.

***Issue 5. Missing information in the Method***

In agreement with reviewer’s suggestions, PACS has now been clearly described as being a measure previously validated to the Portuguese population. To our knowledge, there is no validation of the AUDIT questionnaire to the Portuguese population, notwithstanding we followed the translation and the values suggested by *Serviço de Intervenção nos Comportamentos Aditivos e nas Dependências* (2013), which is one of the most regarded institutions in the field of substance abuse in Portugal. Additionally, information specifying the use of verbal informed consent was added.

As described by reviewer 1, the method used to identify the number of factors in the exploratory step was, indeed, Eigenvalue (i.e., a factor was considered when its Eigenvalue was higher than 1), and this information has now been described in the manuscript. Likewise, we followed the reviewer’s advice and performed a Parallel Analysis. Results from this analysis were identical to those obtained with Eigenvalue (see Table 1), in a sense that the same number factors met the selection criteria (i.e., having an Eigenvalue in the Exploratory Factor Analysis superior to the random generated one). We decided not to include the Parallel Analysis in the final version of the manuscript due to a possible redundance with the Eigenvalue approach. However, we highly value the reviewer’s suggestion and would gladly make further changes on this matter if the reviewer deemed necessary.

**Table 1. Parallel Analysis – Comparison between EFA Eigenvalues and randomly generated ones**

| **Factor** | **Eigenvalues** | |
| --- | --- | --- |
|  | **Exploratory Factor Analysis** | **Randomly Generated Parallel Analysis** |
| 1 | 4.628 | 1.293 |
| 2 | 1.453 | 1.207 |
| 3 | 1.201 | 1.162 |
| 4 | 0.762 | 1.118 |
| 5 | 0.710 | 1.069 |
| 6 | 0.624 | 1.035 |
| 7 | 0.585 | 1.007 |
| 8 | 0.538 | 0.967 |
| 9 | 0.456 | 0.933 |
| 10 | 0.413 | 0.896 |
| 11 | 0.360 | 0.860 |
| 12 | 0.269 | 0.819 |

***Minor Issues.***

As suggested by the reviewer, items were checked for double loading and it was accordingly described in the new version of the manuscript (pp. 8, line 172): “The items were checked for double loading to a factor (i.e., having a loading > 0.60 to more than one factor)”. Also, as suggested by the reviewer, the two validity sections were combined under a single header called “Validity”.

**Reviewer 2**

We thank the reviewer for the overall positive evaluation of the manuscript and for their meaningful comments, that we have carefully tried to address below. Additionally, and as suggested, the whole manuscript was revised by an independent person with a high degree of proficiency in English.

***Issue 1. Writing corrections***

1. In the Abstract, “administrated” has been changed to “administered”.
2. In the Introduction, “However, this type of scales is usually” has been changed to “However, this type of scale is usually”.
3. On page 5 where it showed “67,3%” has been changed to “67.3%”, in accordance with the correct system of decimals. All other decimals have been checked. A zero has been added in front of all decimals.
4. In Data Analysis, “To this extend” has been changed to “To this extent”.
5. In the Results, sentences beginning with “Significant correlation” have been changed to “A significant correlation”.
6. In the Discussion, “contrarily” has been changed to “contrary”.

***Issue 2. Missing description of “risky drinkers” and “non/low risk drinkers” prior to Table 1***

As suggested, it has been stated earlier in the Methods section (pp. 6, line 111) the classification of individuals regarding their pattern of alcohol use. Thus, the meaning of “risky drinkers” and “non/low risk drinkers” has been explicitly described prior to Table 1 appearance.

In this sense the following was added to the manuscript “Additionally, the sample was divided in two groups based on alcohol use pattern, i.e., non/low drinkers (AUDIT < 8) and risky drinkers (AUDIT ≥ 8).”

***Issue 3. Limitation regarding disproportionate number of women to men was not discussed***

As pointed out by Reviewer 2, this limitation is, indeed, important and should be thoroughly discussed. As such, we added information stating that “In addition, the fact that this study presents a significantly higher number of females than males (67.3% vs 32.7%), might be affecting our results. Thus, additional studies trying to match the gender distribution should be conducted in order to validate the present results.”

**References**

Serviço de Intervenção nos Comportamentos Aditivos e nas Dependências. AUDIT - Alcohol Use Disorders Identification Test. Serviço de Intervenção Nos Comportamentos Aditivos e Nas Depências. 2013. Available from: http://www.sicad.pt/PT/Intervencao/RedeReferenciacao/SitePages/detalhe.aspx?itemId=2&lista=SICAD_INSTRUMENTOS&bkUrl=/BK/Intervencao/RedeReferenciacao/
