# Supplementary material for: Portuguese validation of the Alcohol Craving Questionnaire–Short Form–Revised
Source: PLoS One. 2021 May 24;16(5):e0251733. doi: 10.1371/journal.pone.0251733 (PMC8143387; doi:10.1371/journal.pone.0251733)
Supplement: Attachment — Submitted filename: Response to Reviewers.docx [file pone.0251733.s004.docx]

Dear Dr. Wen-Jun Tu,

We sincerely thank you for inviting us to further enhance our manuscript taking into account the reviewer’s recent suggestions. We have considered each suggestion very carefully, and we have rephrased the parts of the manuscript pointed out by them, as specified below. We believe that all the referee’s concerns have been addressed in this new version of the manuscript, and we hope it can now be considered suitable for publication on PLOS ONE.

Please, do not hesitate to contact us for further information or if any other questions come up.

Thank you for your time and consideration.

We look forward to hearing from you.

We thank the reviewer for their useful suggestions. In the next pages, we try to address each of their considerations.

**Reviewer 1**

First of all, we thank the reviewer for their valuable comments and suggestions, specifically the care they gave into writing and grammar corrections and suggestions, as we believe they have helped substantially to improve the manuscript.

***Issue 1. Writing corrections***

1. On page 4, “composed by” was changed to “composed of”; “its likely to be beneficial in measuring craving in multifactorial scales, which can have” was changed to “it is likely to be beneficial to use multifactorial scales for measuring craving, since these can have”; “is usually extensive and with a great number of items” was changed to “usually has a great number of items”; “negative effect on respondents’ motivational and cognitive processes” was changed to “negative effect on respondents’ willingness to accurately complete them”; and “short-forms of multifactorial alcohol” was changed to “short forms of multifactorial alcohol”.
2. On page 6, “non/low drinkers” was changed to “non/low-risk drinkers”. This changed was also conducted in every instance the consumption group was named.
3. Further on page 6, “is a self-reported measure” was changed to “is a self-report measure”, and this correction was applied to every instance of the same error; “12–item” was changed to “12 items”: “Items are ranked on a” was changed to “Items are rated on a”; “same four factors than ACQ-Now” was changed to “same four factors as the ACQ-Now”; and “the intent and planning to drink” was changed to “the intention and plan to drink”.
4. On page 7, “restrain capacity” was changed to “restraint capacity”; and “validated to European Portuguese speakers” was changed to “validated for European Portuguese speakers”.
5. On page 8, “inter-items correlation” was changed to “inter-item correlation”.
6. On page 9, “Additionally, using the two groups based on alcohol use pattern (see Table 1), it was conducted a repeated measures ANOVA” was changed to “Additionally, in order to compare ACQ-SF-R scores for participants in the two levels alcohol risk (see Table 1), a repeated measures ANOVA was conducted”; and “were assessed” was clarified and changed to “were conducted separately within each gender”.
7. On page 10, “comprised by items” was changed to “comprised of items”; and “double loaded to any factor” was changed to “double loaded on any factor”.
8. On page 12, “When assessing groups by gender” was changed to “When conducting EFAs separately within each gender”; and “for F2 (Purposefulness) instead of F3” was changed to “on F2 (Purposefulness) than F3”.
9. On page 13, “Likewise, mean inter-item correlation” was written using its plural “Likewise, mean inter-item correlations”; “the ACQ-SF-R possesses good psychometric properties and reveals to be a valid and reliable measure” was changed to “the ACQ-SF-R possesses good psychometric properties and is a valid and reliable measure”; “showed cohesiveness between genders” was changed to “showed cohesiveness within each gender”; “validation” when referring to the previously validated versions of the scale was changed to “validations” since its plural form should be the one used; “Thus, the present study seems to complement the PACS” was changed to “Thus, the present study seems to complement the psychometric results found for the Portuguese version of the PACS”; and “which to date is the only questionnaire validated in European Portuguese assessing alcohol craving” was changed to “which to date is the only questionnaire assessing alcohol craving validated in European Portuguese”.
10. On page 14, “satisfactory concurrent validity between ACQ-SF-R and AUDIT” was changed to “satisfactory concurrent validity between the ACQ-SF-R and the AUDIT”; given its redundancy “dimensional” was removed from “four-factor dimensional measure”.
11. On page 15, “reflecting the intent and planning to drink alcohol” was changed to “reflecting the intention and plan to drink”; “and, consequently, low purposefulness to drink” was changed to “and, consequently, scored low on the scale of purposefulness in regard to drinking”; “Despite the sample size is higher” was changed to “While a strength of the study was that the sample size was higher”; and “than previous validations of the scale” was changed to “than previous validation studies of the scale”.
12. On page 16, “match the gender distribution” was changed to “equate the number of each gender and include a general adult population”, and the following sentence regarding greater sociodemographic representation was removed; “it allows to assess” was changed to “it allows assessment of”.

***Issue 2. Table 1 was missing the necessary note defining the abbreviations used***

We thank the reviewer for bringing out attention to this issue. The following note was added below Table 1 “Note: M, mean; SD, standard deviation; AUDIT, Alcohol Use Disorders Identification Test; PACS, Penn Alcohol Craving Scale; ACQ-SF-R, Alcohol Craving Questionnaire – Short Form – Revised.”; the remaining tables were checked for abbreviations that were not correctly described.

***Issue 3. The AUDIT was not correctly described has being translated to European Portuguese***

In agreement with the reviewer’s suggestion, we added the following sentence to our Measures on page 7 “In the present study, we used the European Portuguese translated version of the AUDIT”, hence, clarifying the scales translation into European Portuguese.

***Issue 4. Information regarding EFA characteristics was mentioned in the Method and repeated in the Results***

Following the reviewer’s suggestion, duplicated information describing the EFA was removed from the Results. Additionally, the sentence that mentioned the use of ULS in our EFA was moved so that it gets better highlighted during the reading of the manuscript.

***Issue 5. Table 5 removal***

As highlighted by the reviewer, it is customary to take the authors’ word for possible changes in the reliability of the scale when removing any items. As such, Table 5 was considered by us redundant and consequently removed from the manuscript.

***Issue 6. No information (means and standard deviation) regarding the significant differences in craving between the two consumption groups were reported***

We would like to thank the reviewer for alerting us of this issue. Considering their suggestion, we added the following table to the manuscript following the Validity section of the manuscript.

|  | **Non/Low Risk Drinkers** M(SD)  N = 454 | **Risky Drinkers** M(SD)  N = 129 |
| --- | --- | --- |
| F1 - Emotionality | 1.60 (0.91) | 2.56 (1.40) |
| F2 - Purposefulness | 2.35 (1.45) | 3.83 (1.57) |
| F3 - Compulsivity | 1.34 (0.58) | 2.11 (1.21) |
| ACQ-SF-R – Full Scale | 1.70 (0.73) | 2.72 (1.10) |

**Table 5. Craving scores across alcohol use pattern groups**

Note: ACQ-SF-R, Alcohol Craving Questionnaire – Short Form – Revised; M, mean; SD, standard deviation.

***Issue 7. Too strong wording was used when describing possible uses for the ACQ-SF-R***

We sincerely thank the reviewer for highlighting this issue, and taking into account their suggestions. Thus, in the page 15, the phrase “Consequently, ACQ-SF-R seems to be a useful tool for clinical practice, helping to detect risky drinking, and predict relapses and treatment outcomes” was changed to “Consequently, ACQ-SF-R may be a useful tool for clinical practice, being able to help to detect risky drinking”
